# FX5 as a non-steroidal GR antagonist improved glucose homeostasis in type 2 diabetic mice via GR/HNF4α/miR-122-5p pathway

**DOI:** 10.18632/aging.202275

**Published:** 2020-12-09

**Authors:** Xin Xu, Yidi Chen, Danyang Zhu, Tong Zhao, Rui Xu, Jiaying Wang, Lihong Hu, Xu Shen

**Affiliations:** 1 Key Laboratory of Drug Target and Drug for Degenerative Disease of Jiangsu Province, Nanjing University of Chinese Medicine, Nanjing 210023, China

**Keywords:** type 2 diabetes, glucocorticoid receptor, gluconeogenesis, HNF4α, miR-122-5p

## Abstract

Type 2 diabetes mellitus (T2DM) is a chronic metabolic disease characterized by glucose metabolic disorders, and gluconeogenesis inhibiting is a promisingly therapeutic strategy for T2DM. Glucocorticoid receptor (GR) is tightly implicated in the regulation of gluconeogenesis, although the underlying mechanism remains obscure. Here, we discovered that small molecule, 5-chloro-N-[4-chloro-3-(trifluoromethyl)phenyl]thiophene-2-sulfonamide (FX5) as a new non-steroidal GR antagonist efficiently ameliorated glucose homeostasis in *db/db* and HFD/STZ-induced T2DM mice. The mechanism underlying the suppression of FX5 against gluconeogenesis was highly investigated. FX5 suppressed gluconeogenetic genes G6Pase and PEPCK in mouse primary hepatocytes and liver tissues of T2DM mice. Results of mammalian one-hybrid and transactivation as well as nuclear translocation assays totally evaluated the antagonistic features of FX5 against GR. Moreover, *si*RNA and overexpression related assays verified that FX5 alleviated gluconeogenesis either directly by antagonizing GR or indirectly through GR/HNF4α/miR122-5p signaling pathway. Our work has presented a new mode for GR antagonist in the regulation of gluconeogenesis, which is expected to highlight the potential of FX5 in the treatment of T2DM.

## INTRODUCTION

Type 2 diabetes mellitus (T2DM) is a chronic metabolic disease, which is characterized by glucose and lipid metabolic disorders. Although the pathogenesis of T2DM is complicated, inadequate insulin secretion and insulin resistance in peripheral organs are highly contributable to the progression and development of T2DM [1]. The liver as a major metabolic organ plays a fundamental role in glucose homeostasis [2, 3]. While fasting, hepatic gluconeogenesis and glycogenolysis are stimulated by glucagon resulting in increased level of blood glucose production. However, due to the limited glycogen level in the liver, hepatic gluconeogenesis is often the predominant source for glucose production under prolonged starving [3, 4]. Gluconeogenesis is the process that converts the substrates lactate, glycerol and amino acids into glucose, and it is believed that dysregulation of gluconeogenesis contributes to increased endogenous glucose production and gives rise to fasting hyperglycemia in T2DM patients [2, 5]. In addition, hepatic gluconeogenesis inhibitors like metformin and pioglitazone have been used as first-line clinical medication against T2DM for decades, implying that targeting gluconeogenesis inhibition is a potently therapeutic strategy for T2DM [6, 7].

Hepatic glucose metabolism is modulated by numerous hormones including glucagon, insulin and glucocorticoid [3]. Glucocorticoid is mediated by glucocorticoid receptor (GR), which belongs to a member of nuclear receptor superfamily staying in cytoplasm as a complex with heat shock proteins (HSPs) like HSP90 and HSP70 in the absence of ligand [8]. When ligand binds to GR, the receptor-ligand complex translocates to the nucleus, causing GR to bind glucocorticoid responsive element (GRE) for regulating downstream gene transcription [9]. Actually, GR has been a credible drug target for decades and its agonists are usually clinically used to treat asthma, skin disorders and rheumatoid arthritis [10].

It was reported that the level of GR expression is abnormally high in diabetic mice and liver-specific deletion of GR improves hyperglycemia, which thus suggests that excess activation of GR might contribute to the pathogenesis of T2DM [11, 12]. In addition, further studies have also illustrated that GR may regulate hepatic gluconeogenesis by binding to GRE in the promoters of gluconeogenic genes glucose-6-phosphatase (G6Pase) and phosphoenolpyruvate carboxykinase (PEPCK) [13, 14]. Besides, GR also indirectly affects gluconeogenic gene transcription by modulating its downstream genes nuclear factor-4α (HNF4α), peroxisome proliferator-activated receptor gamma coactivator-1α (PGC-1α) and forkhead box protein O1 (FoxO1), all of which function potently in the regulation of gluconeogenic genes [15].

Actually, it is due to the GR agonism-mediated gluconeogenesis activation that treatment of GR agonist like dexamethasone (Dex) clinically may cause increased gluconeogenesis and hyperglycemia, which makes the limitation of GR activator usage [16, 17]. In contrast, treatment of diabetic mice or patients with mifepristone (MIFE), a steroidal GR antagonist, could reduce hepatic glucose production by inhibiting the expression of gluconeogenic genes, further implying the beneficial of GR antagonism in improving hyperglycemia [18, 19]. However, long-term use of mifepristone resulted in side effects like hypercortisolemia and adrenal insufficiency [20]. Thus, it is valuable to design non-steroidal GR antagonists with new functional modes to treat T2DM [21, 22].

HNF4α belongs to a member of nuclear receptor superfamily and is a highly conserved transcription factor playing an important role in regulating differentiation, growth and function of hepatocytes [23]. It was reported that HNF4α activates the expressions of G6Pase and PEPCK by binding to HNF4α-binding cis-elements in their promoters [24]. The crucial role of HNF4α in gluconeogenesis regulation was confirmed by liver-specific HNF4α knockout assay in mice [25]. In addition, micro RNAs (miRNAs) are short non-coding single-stranded RNA molecules (19-22 nucleotides), which are encoded by endogenous genes and involved in post-transcriptional gene expressions. It was reported that HNF4α regulates miR122-5p expression in T2DM [26, 27]. In fact, miR122 was perceived as a liver-specific miRNA constituting 70% of the total miRNA population in the liver [28]. Published results have indicated that miR122 is tightly linked to multiple pathophysiology and physiology processes including lipid metabolism, hepatocellular carcinoma and gluconeogenesis [26, 29, 30].

**Figure 1:**
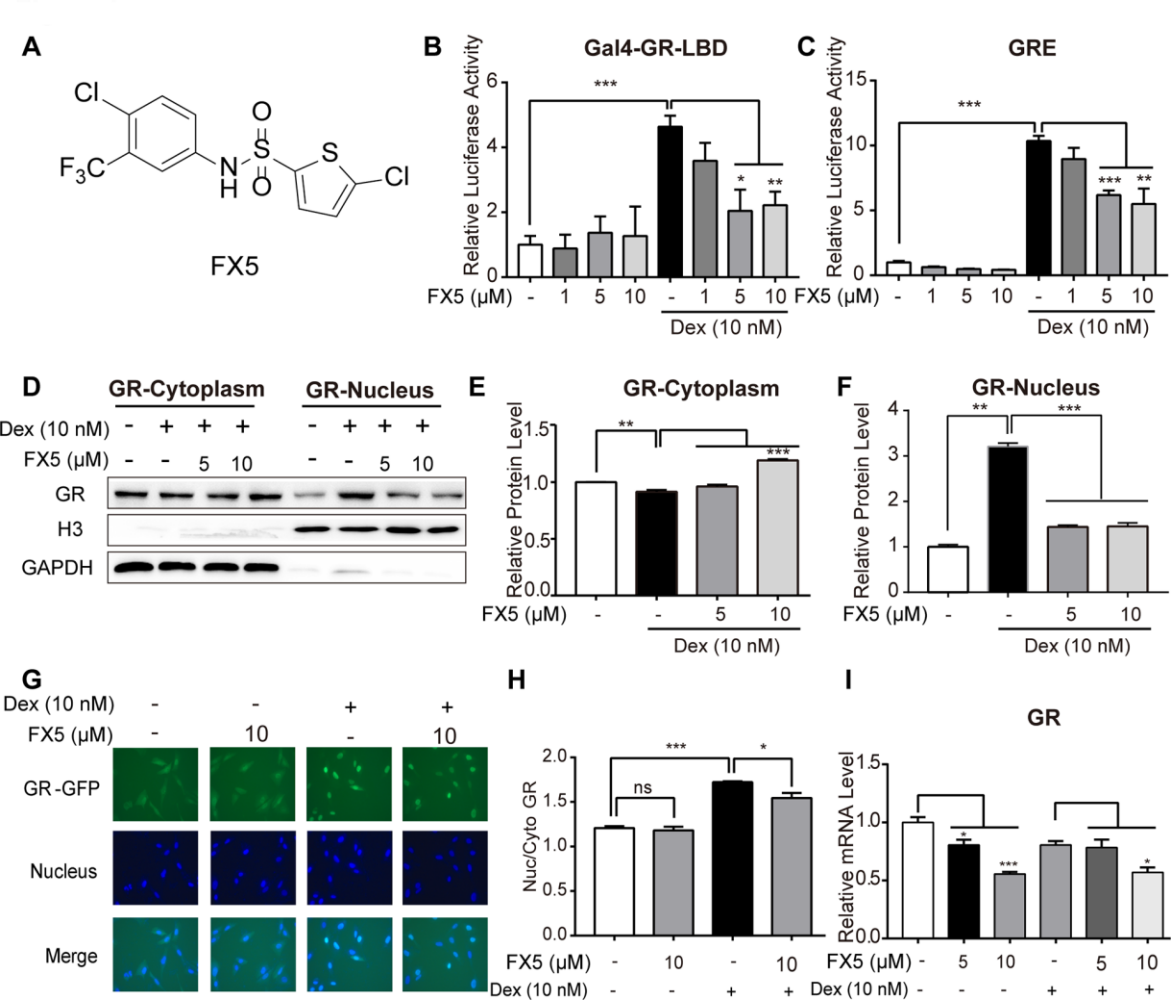
**FX5 was a GR antagonist.** (**A**) Chemical structure of FX5. (**B**) Mammalian one-hybrid assay was carried out to detect the antagonistic activity of FX5 against GR. In the assay, HEK293T cells were co-transfected with the plasmids of pCMX-Gal4-GR-LBD, pUAS-TK-luc and pRL-SV40, and then treated with Dex (10 nM) and FX5 (1, 5 and 10 μM) for 12 h, while luciferase activity was finally measured. (**C**) HEK293T cells were co-transfected with plasmids of pCI-nGFP-GR (C656G), pGL3-GRE-Luc and pRL-SV40, and then incubated with 10 nM Dex and different concentrations of FX5 (1, 5 and 10 μM) for 12 h. Finally, transactivation activity of GR was measured by luciferase reporter assay. (**D**) Mouse primary hepatocytes were treated with Dex (10 nM) and FX5 (5 and 10 μM) for 6 h, and GR protein levels in cytoplasm and nucleus were then measured by western blot assay. Quantification results of cytoplasmic GR protein level to GAPDH (**E**) and nuclear GR protein level to Histone H3 (**F**). (**G**) FX5 inhibited the Dex-stimulated GR-GFP nuclear translocation. (**H**) Ratio of GFP fluorescence intensity of nuclear and cytoplasm. (**I**) Mouse primary hepatocytes were treated with Dex (10 nM) and different concentrations of FX5 (1, 5 and 10 μM) for 6 h, and mRNA levels of GR were measured by quantitative RT-PCR analysis. Values were mean ± S.E.M (n=3/group) (*P<0.05, **P<0.01 and ***P<0.001).

Here, we determined that small molecule, 5-chloro-N-[4-chloro-3-(trifluoromethyl)phenyl]thiophene-2-sulfonamide (FX5, Figure 1A), as a non-steroidal GR inhibitor effectively reduced gluconeogenesis and improved glucose homeostasis in *db*/*db* and HFD/STZ-induced T2DM mice. The underlying mechanisms have been intensively investigated. FX5 suppressed gluconeogenesis either directly by antagonizing GR or indirectly through GR/HNF4α/miR122-5p signaling pathway. To our knowledge, our work might be the first report on the regulation of GR antagonist against gluconeogenic genes in such a novel mode, which is expected to provide a new approach for developing GR modulators and highlight the potential of FX5 in the treatment of T2DM.

## RESULTS

### FX5 was a GR antagonist

### FX5 inhibited GR transactivation activity

GR as a nuclear receptor is structurally composed of a poorly conserved amino-terminal domain (NTD), a highly conserved DNA-binding domain (DBD) and a ligand-binding domain (LBD) [31]. In the current work, mammalian one-hybrid assay system [32] was constructed based on GR-LBD, and mammalian transactivation assay system [33] was established on the strength of glucocorticoid response elements (GRE) located in the regulation regions of GR-target genes for detection of GR binders with potentially agonistic or antagonistic activity against GR.

To find GR antagonists, we at first performed mammalian one-hybrid and transactivation assays against the lab in-house small compound library to screen out the active compounds able to antagonize GR, and FX5 (Figure 1A) was finally determined for its better inhibitory activity. As indicated in Figure 1B, 1C, FX5 effectively antagonized the Dex-induced activation of either reporter gene expression in mammalian one-hybrid assay or luciferase report gene expression in mammalian transactivation assay. These results thus demonstrated that FX5 displayed antagonistic activity against GR.

### FX5 suppressed Dex-stimulated GR nuclear translocation and mRNA level of GR

According to the published reports, GR as a transcription factor shuttles between cytosol and nucleus [34] and translocates from cytosol to nucleus for regulating downstream genes when cell is stimulated by GR agonist like Dex [8]. In contrast, GR antagonist inhibits GR translocation [33]. With these facts, we detected the potential effect of FX5 on GR intracellular distribution. In the assay, we separated the cytoplasmic and nuclear proteins from the Dex or FX5-treated mouse primary hepatocytes and found that FX5 efficiently restrained the Dex-stimulated GR nuclear translocation (Figure 1D–1F). In addition, U2OS/GR-GFP stable cell was also used to detect the influence of FX5 on GR distribution. As indicated in Figure 1G, 1H, FX5 itself had no effects on GR cellular distribution, but could inhibit the Dex-stimulated GR nuclear translocation. Moreover, the results in Figure 1I demonstrated that FX5 decreased mRNA expression of GR, which indicated that FX5 could suppress GR transcription in mouse primary hepatocytes.

Collectively, all results suggested that FX5 was an antagonist of GR.

### FX5 reduced hepatic glucose production and gluconeogenesis

Since GR antagonist has an activity in inhibiting gluconeogenesis [35], we testified the potential of FX5 in suppressing glucose production and gluconeogenic gene expression in mouse primary hepatocytes.

### FX5 inhibited Dex-induced gluconeogenesis

In the assay, we at first detected the potential toxicity of FX5 against mouse primary hepatocytes and found that FX5 had no impacts on hepatocytes viability in 1, 5 or 10 μM (Supplementary Figure 1A). Thus, we chose FX5 at these concentrations for the following experiments.

**Figure 2:**
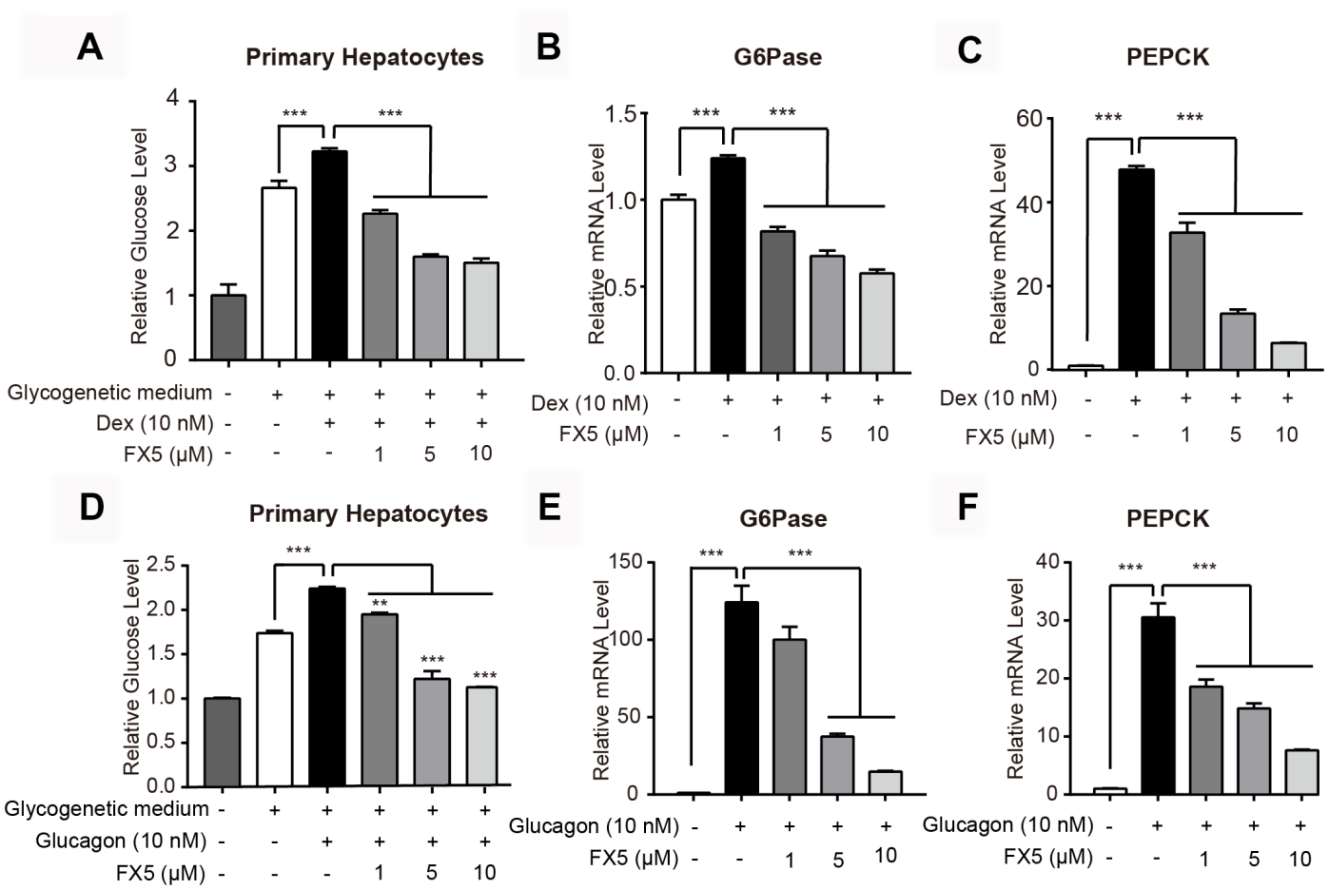
**FX5 inhibited Dex or glucagon-stimulated gluconeogenesis.** (**A**) Mouse primary hepatocytes were pretreated with Dex (10 nM) for 12 h, followed by incubation with Dex and FX5 (1, 5 and 10 μM) for 6 h in glycogenetic medium. Glucose concentration in the medium was detected to evaluate glucose output level. (**B**, **C**) Mouse primary hepatocytes were treated with Dex (10 nM) and different concentrations of FX5 (1, 5 and 10 μM) for 6 h, and mRNA levels of G6Pase and PEPCK were measured by quantitative RT-PCR analysis. (**D**) Glucose production assay was conducted in mouse primary hepatocytes. Cells were pretreated with glucagon (10 nM) for 12 h, and then cultured for another 6 h in glycogenetic medium with glucagon and different concentrations of FX5 (1, 5 and 10 μM). Finally, glucose level in the medium was measured. (**E**, **F**) Mouse primary hepatocytes were treated with glucagon (10 nM) and different concentrations of FX5 (1, 5 and 10 μM) for 6 h, and mRNA levels of G6Pase and PEPCK were detected by qRT-PCR analysis and normalized to GAPDH. All results were presented as mean ± S.E.M (n=3/group) (**P<0.01 and ***P<0.001).

As shown in Figure 2A, FX5 dose-dependently reduced the Dex-increased glucose production in mouse primary hepatocytes. Next, the impact of FX5 on the two major gluconeogenic genes G6Pase and PEPCK was detected by quantitative RT-PCR (qRT-PCR) assay, and the results (Figure 2B, 2C) demonstrated that FX5 effectively lessened the Dex-enhanced mRNA levels of G6Pase and PEPCK. Therefore, these results indicated that FX5 restrained the Dex-induced gluconeogenesis.

### FX5 suppressed glucagon-stimulated gluconeogenesis

Considering that glucagon is a major stimulus of gluconeogenesis and hyperglucagonemia is present in poorly controlled diabetes [36], we incubated glucagon (10 nM) with hepatocytes trying to mimic pathological state of T2DM, and the impact of FX5 on glucose output and gluconeogenic gene expression were then detected.

As shown in Figure 2D–2F, FX5 dose-dependently reduced the glucagon-enhanced glucose production and inhibited the glucagon-induced mRNA levels of G6Pase and PEPCK in mouse primary hepatocytes. Thus, all results indicated that FX5 suppressed the glucagon-stimulated gluconeogenesis.

### FX5 suppressed gluconeogenesis by targeting GR

Given that FX5 has been determined a GR antagonist capable of inhibiting gluconeogenesis, GR antagonist mifepristone (MIFE) was next applied to confirm the GR-targeted effect of FX5 on gluconeogenesis.

**Figure 3:**
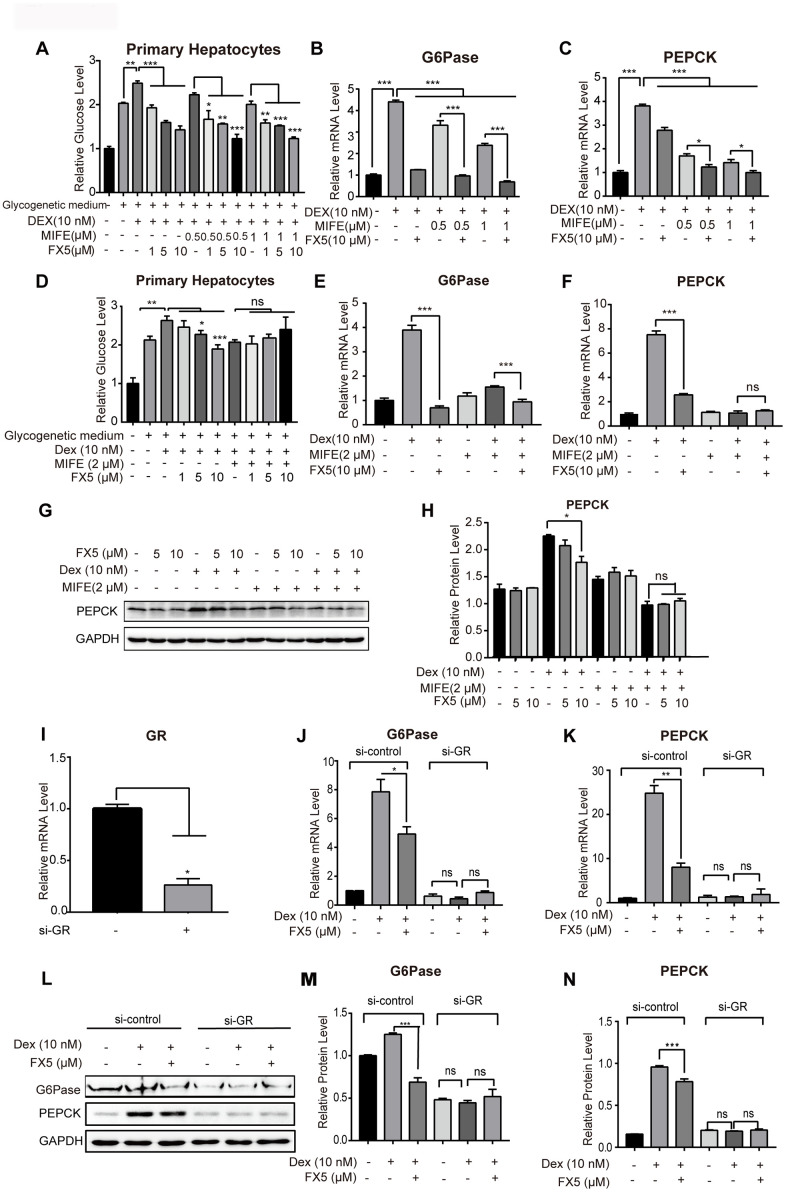
**FX5 suppressed gluconeogenesis by antagonizing GR.** (**A**) Glucose production assay was conducted in mouse primary hepatocytes. Cells were pretreated with glucagon (10 nM) and different concentration of mifepristone (MIFE) (0.5 and 1 μM) for 12 h, and then cultured for another 6 h in glycogenetic medium with glucagon and different concentrations of FX5 (1, 5 and 10 μM). Finally, glucose level in the medium was measured. (**B**, **C**) Mouse primary hepatocytes were treated with 10 nM Dex, MIFE (0.5 or 1 μM) and 10 μM FX5 for 6 h. Then, mRNA levels of G6Pase and PEPCK were measured by qRT-PCR. (**D**) Primary hepatocytes were pretreated with Dex (10 nM), mifepristone (MIFE) (2 μM) and different concentrations of FX5 (1, 5, 10 μM). Glucose level in the medium was measured. (**E**, **F**) Mouse primary hepatocytes were treated with 10 nM Dex, 2 μM MIFE and 10 μM FX5 for 6 h. Then, the effect of FX5 on expressions of G6Pase (82% to 39%) and PEPCK (66% to no significance) were repressed by MIFE by quantitative RT-PCR assay. (**G**) Mouse primary hepatocytes were incubated with 10 nM Dex, 2 μM MIFE and different concentrations of FX5 (5, 10 μM) for 6 h, and protein levels of PEPCK and GAPDH were measured by western blot and quantified in (**H**). (**I**–**N**) Mouse primary hepatocytes were transfected with *si*-control or *si*-GR for 48h. Hepatocytes were then treated with 10 nM Dex and 10 μM FX5 for 6 h. (I) The mRNA interference efficiency level of GR by *si*-GR was 73.6%. mRNA levels of (**J**) G6Pase and (**K**) PEPCK were measured by quantitative RT-PCR. (**L**) Protein levels of G6Pase, PEPCK and GAPDH were measured by western blot assay. Quantification of (**M**) G6Pase and (**N**) PEPCK protein levels. All results were normalized to GAPDH. Values were mean ± S.E.M (n=3/group) (*P<0.05, **P<0.01 and ***P<0.001).

In the assay, primary hepatocytes were treated with different concentrations of GR antagonist MIFE (0.5, 1 μM) and FX5 (1, 5, 10 μM). As indicated in Figure 3A, either FX5 or MIFE dose-dependently reduced glucose level and FX5 antagonized the activity of MIFE in inhibiting the level of glucose. Moreover, incubation of FX5 with MIFE rendered stronger inhibitory effects on the expression of gluconeogenesis related gene G6Pase (Figure 3B) and PEPCK (Figure 3C) compared with the treatment of either FX5 or MIFE.

Next, MIFE was applied to investigate whether the inhibitory effect of FX5 on glucose production was through antagonizing GR. As indicated in Supplementary Figure 1B, MIFE dose-dependently inhibited the Dex-stimulated glucose level in primary hepatocytes, while such an inhibitory effect reached a maximum value by treatment of MIFE at 2 μM as indicated by the finding that even MIFE at 3 μM failed to further suppress the Dex-stimulated glucose level. With these facts, we assessed the effect of MIFE at 2 μM on the suppression of FX5 against glucose production. As shown in Figure 3D, FX5 antagonized the Dex-stimulated hepatic glucose production, and MIFE (2 μM) treatment obviously deprived FX5 of such an antagonistic activity, which indicated that FX5 inhibited glucose production via antagonizing GR.

For more details, we investigated the effect of FX5 on the expressions of G6Pase and PEPCK by qRT-PCR assay. As illustrated in Figure 3E, 3F, MIFE repressed the efficiency of FX5 in inhibiting the Dex-stimulated gene expressions of G6Pase (82% to 39%) and PEPCK (66% to no significance). Additionally, we also detected the influence of FX5 on PEPCK at protein level. As shown in Figure 3G, 3H, either MIFE or FX5 could efficiently antagonize the Dex-stimulated PEPCK protein level, but MIFE treatment (2 μM) deprived FX5 of its capability in reversing the Dex-induced PEPCK protein level.

Furthermore, *si*-GR based assay was also carried out to verify that FX5 inhibited G6Pase and PEPCK by targeting GR. The result in Supplementary Figure 3A indicated the high transfection efficiency of *si*-RNA as evaluated by fluorescence microscope with FAM-*si*-negative control. Next, we treated primary hepatocytes with *si*-GR for 48 h, and the result (Figure 3I) indicated that *si*-GR exhibited a high interference efficiency in reducing GR gene expression by 73.6%. As indicated in Figure 3J–3N, *si*-GR not only deprived Dex of its capability in stimulating the expression of gluconeogenic genes (G6Pase and PEPCK) but also blocked the activity of FX5 in antagonizing the Dex-stimulated protein expressions of G6Pase and PEPCK.

Additionally, considering that 11β-hydroxysteroid dehydrogenase 1 (11β-HSD1) expression is increased in T2DM [12] and 11β-HSD1 converts inactive 11-dehydrocorticosterone into bio-active corticosterone thus amplifying GR signaling in hepatocytes [37, 38], we investigated the potential impact of FX5 on 11β-HSD1. As shown in Supplementary Figure 1C–1E, FX5 suppressed the Dex-stimulated protein expression of PEPCK but rendered no influence on the protein level of 11β-HSD1, implying that 11β-HSD1 was not involved in the regulation of FX5 against GR.

Finally, given that glucagon as a diabetogenic hormone stimulates gluconeogenesis mainly via cAMP/PKA/CREB pathway [39, 40] and FX5 suppressed glucagon-induced stimulation of glucose output and gluconeogenic genes, we inspected whether FX5 regulated glucagon-mediated PKA/CREB signaling in primary hepatocytes by western blot assay. As indicated in Supplementary Figure 1F, 1G, FX5 rendered no influence on the glucagon-induced protein level of p-PKA or p-CREB, thereby indicating that FX5 suppressed gluconeogenesis independent of PKA/CREB pathway.

Together, all above-mentioned results indicated that FX5 inhibited gluconeogenesis by antagonizing GR.

### FX5 inhibited gluconeogenic genes via GR/HNF4α/miR122-5p signaling pathway in primary hepatocytes

### FX5 reduced HNF4α gene expression in primary hepatocytes

As indicated in the published reports, GR directly binds to GRE sequence in the promoters of G6Pase and PEPCK [41], and HNF4α may regulate G6Pase and PEPCK gene expressions [42]. In the liver, GR binds to HNF4α distal enhancer and enhances transcription of *hnf4α* gene thus activating gluconeogenic genes [46]. With these facts, we investigated the potential of FX5 in regulating HNF4α gene expression.

**Figure 4:**
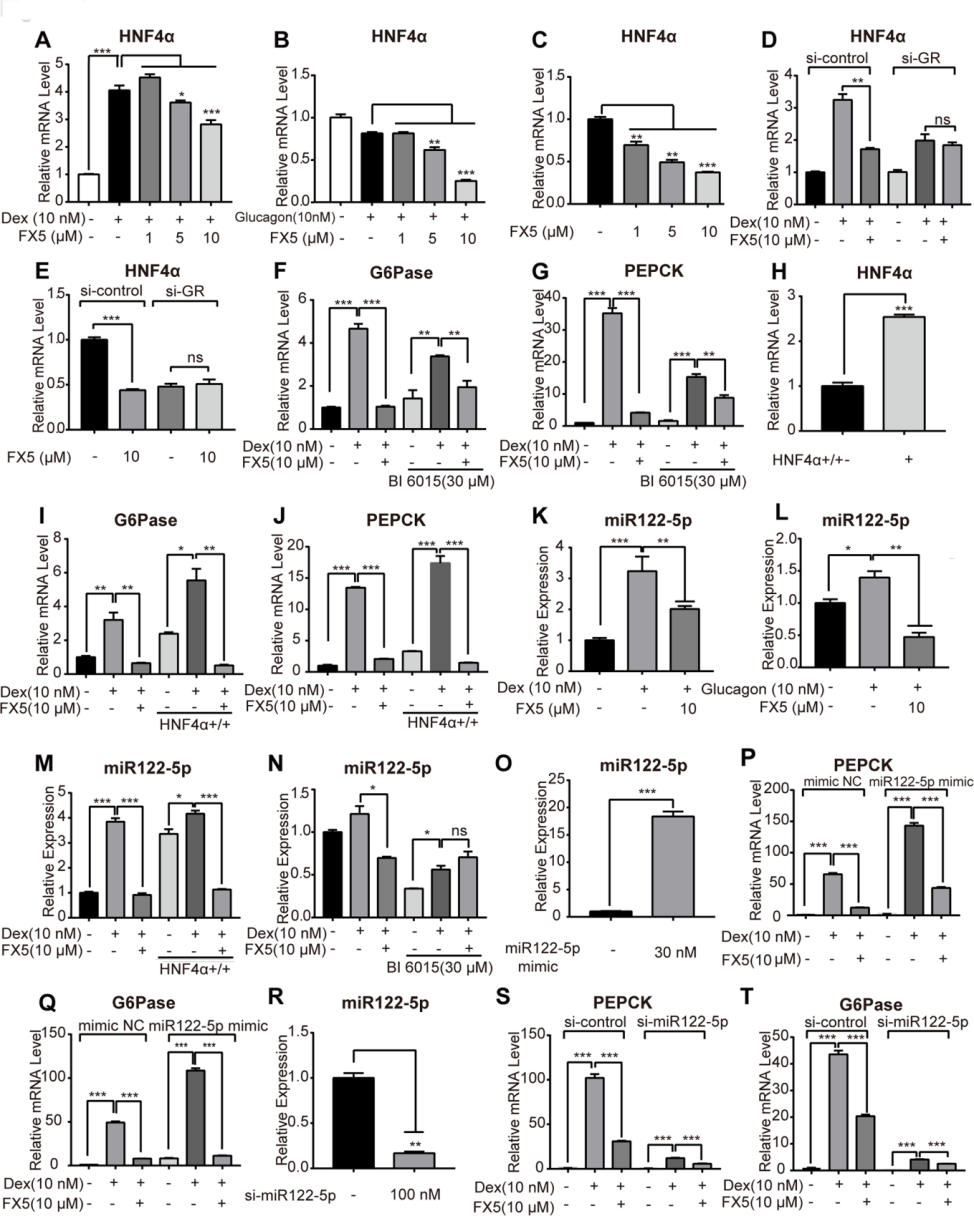
**FX5 suppressed gluconeogenesis via GR/HNF4α/miR122-5p pathway in hepatocytes.** Mouse primary hepatocytes were treated with different concentrations of FX5 (1, 5, 10 μM) and (**A**) 10 nM Dex or (**B**) 10 nM glucagon for 6 h, and mRNA level of HNF4α was investigated by qRT-PCR assay. (**C**) Mouse primary hepatocytes were treated with FX5 (1, 5, 10 μM) for 6 h, HNF4α mRNA level was then detected. (**D**, **E**) Mouse primary hepatocytes were transfected with *si*-control or *si*-GR for 48h. Hepatocytes were then treated with 10 nM Dex and 10 μM FX5 or 10 μM FX5 for 6 h. mRNA level of HNF4α was measured. (**F**, **G**) Mouse primary hepatocytes were treated with 30 μM BI 6015 for 24 h, then incubated with 10 nM Dex and 10 μM FX5 for 6 h. The suppression mRNA levels of (**F**) G6Pase (77.8% to 44.3%) and (**G**) PEPCK (88.2% to 44.9%) in the presence of BI6015 were measured by quantitative RT-PCR. (**H**–**J**) Mouse primary hepatocytes were transfected with HNF4α overexpression plasmid for 48h. Hepatocytes were then treated with 10 nM Dex and 10 μM FX5 for 6 h. mRNA level of (**H**) HNF4α, (**I**) G6Pase and (**J**) PEPCK were measured by quantitative RT-PCR. (**K**, **L**) Mouse primary hepatocytes were incubated in the presence of (**K**) 10 nM Dex or (**L**) 10 nM glucagon with 10 μM FX5 and miR122-5p level was measured by quantitative RT-PCR. (**M**) Mouse primary hepatocytes were transfected with HNF4α overexpression plasmid and then treated with 10 nM Dex and 10 μM FX5 for 6 h. Expression level of miR122-5p was measured by quantitative RT-PCR. (**N**) Mouse primary hepatocytes were treated with 30 μM BI6015 for 24 h, then incubated with 10 nM Dex and 10 μM FX5 for 6 h. Expression level of miR122-5p was quantified. (**O**-**Q**) Mouse primary hepatocytes were transiently transfected with micrON mimic NC #22 or micrON mimic NC #22 (5 FAM) for 48h and then were treated with 10 nM Dex and 10 μM FX5 for 6 h. Expression levels of (**O**) miR122-5p, (**P**) PEPCK and (**Q**) G6Pase were measured by qRT-PCR. (**R**–**T**) Mouse primary hepatocytes were transiently transfected with *si*-control or *si*-miR122-5p for 48h. Hepatocytes were then treated with 10 nM Dex and 10 μM FX5 for 6 h. Expression levels of (**R**) miR122-5p (83.2% interference efficiency), (**S**) PEPCK (70% to 52.5%) and (**T**) G6Pase (53.3% to 38.7%) were measured by quantitative RT-PCR in either absence or presence of *si*-miR122-5p. Values were mean ± S.E.M (n=3/group) (*P<0.05, **P<0.01 and ***P<0.001).

In the assay, we treated mouse primary hepatocytes with Dex or FX5, and HNF4α mRNA level was measured. As shown in Figure 4A, FX5 antagonized the Dex-stimulated HNF4α gene expression. Meanwhile, we also determined that FX5 suppressed HNF4α mRNA level in the presence or absence of glucagon (Figure 4B, 4C). Therefore, all results demonstrated that FX5 could repress HNF4α gene expression in primary hepatocytes.

### *FX5 repressed* gluconeogenic genes *partially through GR/HNF4α signaling in primary hepatocytes*

Next, we attempted to investigate the detailed information relating to the regulation of FX5 against HNF4α in terms of FX5-mediated suppression of gluconeogenesis by targeting GR in primary hepatocytes. As indicated in Figure 4D, 4E, *si*-GR deprived FX5 of its capability in antagonizing the Dex-induced upregulation or basal expression of HNF4α gene. Thus, these results demonstrated that FX5 inhibited mRNA level of HNF4α by targeting GR.

In addition, BI6015 (HNF4α inhibitor) based assay was also carried out. In the assay, BI6015 was incubated with primary hepatocytes for 24 hours and treated with FX5 and Dex for 6 hours, followed by detection of mRNA levels of G6Pase and PEPCK. As indicated in Figure 4F, 4G, either FX5 or BI6015 treatment could antagonize the Dex-induced activation on mRNA level of G6Pase or PEPCK. Notably, in the presence of BI6015 (30 μM), the repression efficiency of FX5 on mRNA levels of G6Pase (77.8% to 44.3%) and PEPCK (88.2% to 44.9%) were decreased, which demonstrated that BI6015 even at high concentration failed to completely block the capability of FX5 in antagonizing the Dex-promoted mRNA level of G6Pase or PEPCK.

To investigate whether FX5 suppressed gluconeogenic genes through HNF4α, HNF4α overexpression plasmid was applied in the assay against primary hepatocytes (The high efficiencies of HNF4α overexpression and transfection were indicated in Figure 4H and Supplementary Figure 3B). As indicated in Figure 4I, 4J, Dex increased mRNA levels of G6Pase and PEPCK in the case of HNF4α overexpression, and FX5 reversed such Dex-stimulated gene expressions. Thus, these results indicated that FX5 antagonized the HNF4α-induced gluconeogenic gene expressions.

Therefore, FX5 suppressed G6Pase and PEPCK genes expression by regulating either GR or GR/HNF4α signaling in primary hepatocytes, in consistence with the published report that GR may directly or indirectly regulate gluconeogenic genes [41].

### FX5 reduced hepatic gluconeogenesis via GR/HNF4α/miR122-5p pathway in primary hepatocytes

Given that miR122-5p is regulated by HNF4α and is involved in gluconeogenic regulation [26], we investigated the potential regulation of FX5 against miR122-5p in primary hepatocytes. As indicated in Figure 4K, 4L, FX5 antagonized Dex or glucagon-induced enhancement on miR122-5p expression. In addition, HNF4α overexpression plasmid and HNF4α inhibitor BI6015 were employed to examine whether FX5 suppressed miR122-5p depending on HNF4α. As shown in Figure 4M, 4N, FX5 suppressed the HNF4α overexpression-induced upregulation of miR122-5p, and BI6015 treatment deprived FX5 of its capability in antagonizing the Dex-induced miR-122-5p expression. These results thus implied that miR122-5p might function in the down-stream of GR/HNF4α signaling.

Since FX5 has been determined to regulate gluconeogenic genes partially through GR/HNF4α signaling, we next verified whether GR/HNF4α/miR-122-5p signaling was partially responsible for FX5-mediated suppression of G6Pase and PEPCK in primary hepatocytes. In the assay, miR122-5p mimic or *si*-miR122-5p was transfected to the cells for up-regulating or down-regulating miR122-5p expression (The results in Supplementary Figure 3C and Figure 4O demonstrated the high efficiencies of transfection and expression of miR122-5p, and the result in Figure 4R showed the high efficiency of interference of miR122-5p expression in cells). With miR122-5p mimic (Figure 4P, 4Q), Dex stimulated mRNA levels of PEPCK and G6Pase, and such stimulations were obviously antagonized by FX5 treatment. As indicated in Figure 4S, 4T, it was interesting to find that FX5 could antagonize the Dex-promoted mRNA level of G6Pase (53.3% to 38.7%) or PEPCK (70% to 52.5%) in either absence or presence of *si*-miR122-5p, although the Dex-promoted level of G6Pase or PEPCK was largely weakened by *si*-miR122-5p. Therefore, these results thus implied that FX5 suppressed gluconeogenic genes G6Pase and PEPCK partially through GR/HNF4α/miR122-5p signaling.

### FX5 treatment improved glucose homeostasis in T2DM mice

As we have determined the capability of FX5 in suppressing gluconeogenesis in mouse primary hepatocytes, we further investigated its potential in improving glucose homeostasis in *db*/*db* and HFD/STZ-induced T2DM mice.

### FX5 ameliorated hyperglycemia of T2DM mice

In the assay, *db*/*db* (n=10/group) and HFD/STZ (n≥7/group) induced T2DM mice were orally administrated with vehicle (Veh) or FX5 (30, 60 mg/kg) once per day for 5 weeks. During the period of administration, food intake and body weight were recorded, and blood glucose level after 6 h fasting was measured every week, while HbA1c level was detected after mice sacrifice.

**Figure 5:**
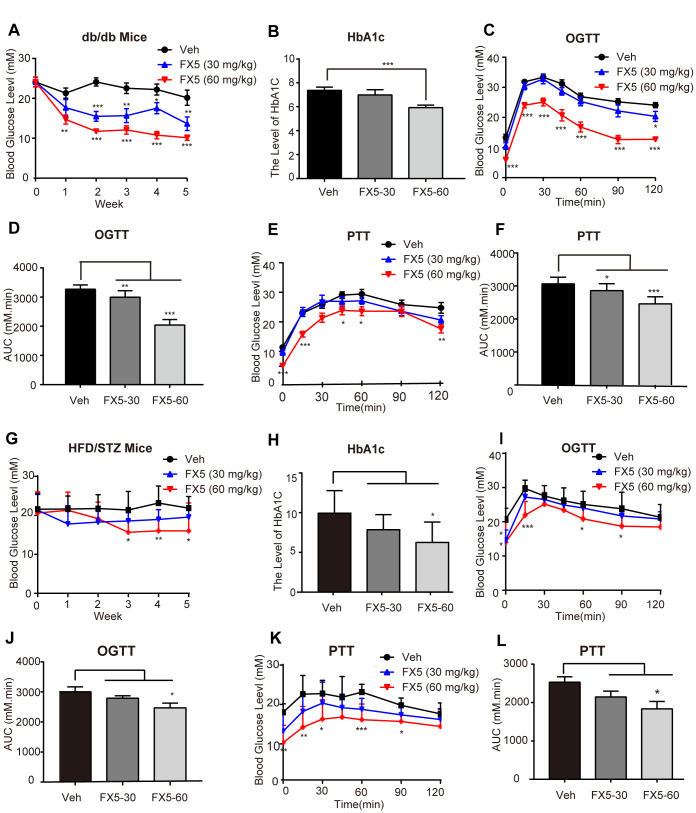
**FX5 treatment improved glucose homeostasis in *db*/*db* and HFD/STZ-induced T2DM mice.** (**A**) *db*/*db* mice were divided into three groups (n=10/group), and treated with vehicle (Veh), FX5 (30 mg/kg) or FX5 (60 mg/kg) once per day for 5 weeks. Plasma glucose concentration after 6 h fasting was measured weekly. (**B**) After 5-week administration, HbA1c level of *db*/*db* mice in three groups was detected. (**C**) OGTT result and (**D**) AUC result of OGTT in *db*/*db* mice. (**E**) PTT result and (**F**) AUC result of PTT in *db*/*db* mice. (**G**) HFD/STZ-induced T2DM mice were divided into three groups (n≥7/group) and treated with vehicle (Veh), FX5 (30 mg/kg) or FX5 (60 mg/kg) for 5 weeks. Plasma glucose concentration after 6 h fasting was measured weekly. (**H**) After 5-week administration, HbA1c level of HFD/STZ mice in three groups was detected. (**I**) OGTT result and (**J**) AUC result of OGTT in HFD/STZ-induced T2DM mice. (**K**) PTT result and (**L**) AUC result of PTT in HFD/STZ-induced T2DM mice. In all line graphs, black for vehicle (Veh), blue for FX5 (30 mg/kg) and red for FX5 (60 mg/kg). Values were shown as mean± S.E.M (*P<0.05, **P<0.01 and ***P<0.001).

It was found that FX5 treatment effectively decreased the levels of plasma glucose concentration and HbA1c in either *db/db* (Figure 5A, 5B) or HFD/STZ-induced T2DM mice (Figure 5G, 5H), while FX5 treatment had no effects on food intake (Supplementary Figure 4A, 4C) and body weight (Supplementary Figure 4B, 4D) of the mice. These results thus demonstrated that FX5 treatment ameliorated hyperglycemia of T2DM mice.

### FX5 treatment improved glucose and pyruvate tolerances of T2DM mice

To further investigate the regulation of FX5 treatment against the whole-body glucose homeostasis and gluconeogenesis, oral glucose tolerance test (OGTT) and pyruvate tolerance test (PTT) were performed against T2DM mice.

OGTT was carried out in the fourth week of FX5 administration in the mice. The results demonstrated that FX5 treatment improved the glucose tolerance in *db*/*db* (Figure 5C, 5D) and HFD/STZ induced (Figure 5I, 5J) T2DM mice. PTT assay was conducted in the fifth week, and the results indicated that FX5 treatment reduced the gluconeogenesis of the liver in T2DM mice (Figure 5E, 5F for *db*/*db* T2DM mice; Figure 5K, 5L for HFD/STZ induced T2DM mice).

### FX5 treatment reduced gluconeogenesis in T2DM mice

**Figure 6:**
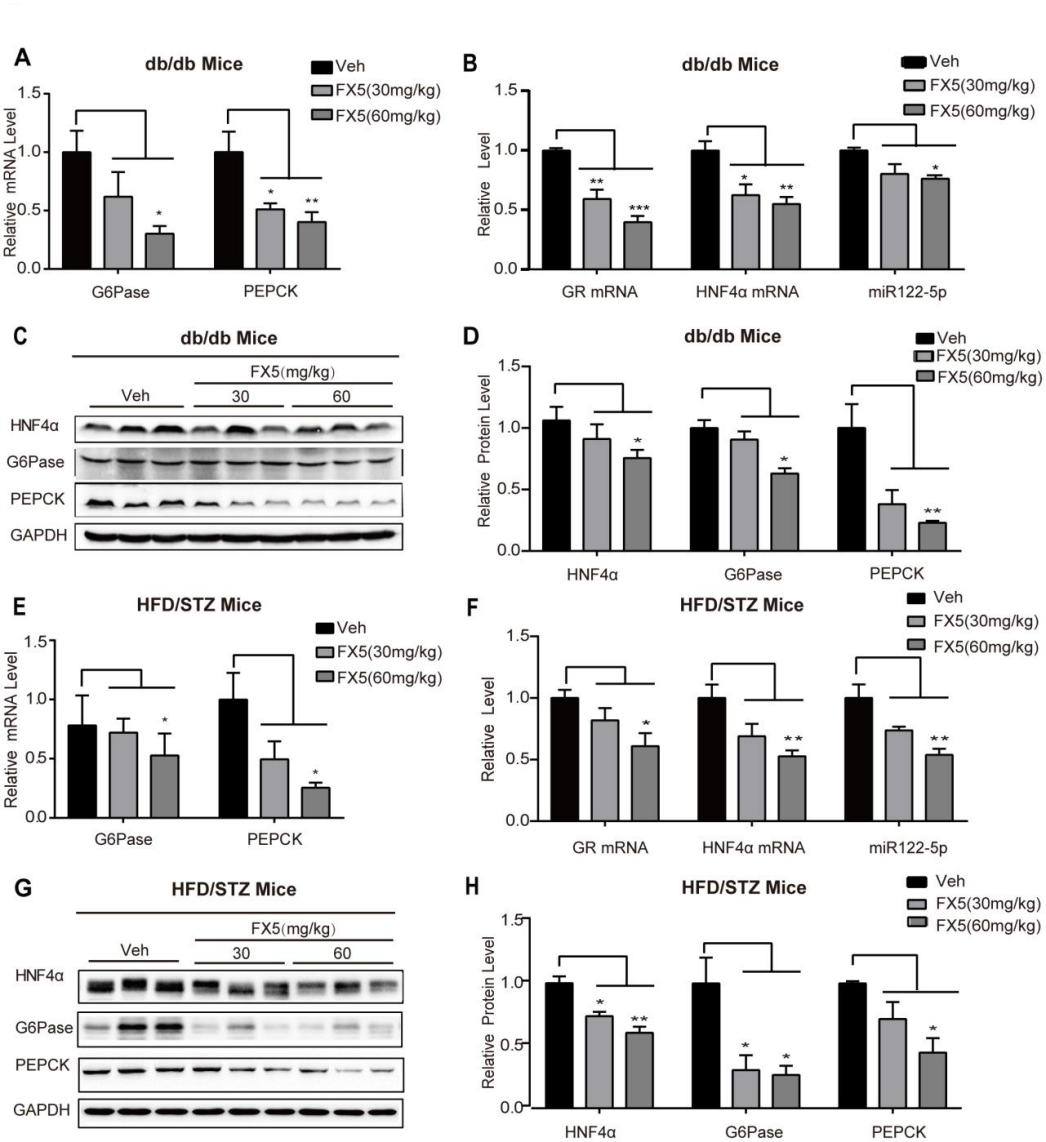
**FX5 treatment suppressed hepatic gluconeogenesis in T2DM mice via GR/HNF4α/miR122-5p pathway.** (**A**) mRNA levels of gluconeogenesis gene G6Pase and PEPCK in the liver tissues of *db*/*db* mice, all results were normalized to GAPDH. (**B**) Expression levels of GR, HNF4α and miR122-5p in the liver of *db/db* mice. (**C**) Protein levels of HNF4α, PEPCK and G6Pase in the liver tissues of *db*/*db* mice were detected by western blot assay and quantified in (**D**). (**E**) mRNA levels of G6Pase and PEPCK in the liver of HFD/STZ-induced T2DM mice, and all results were normalized to GAPDH. (**F**) Expression levels of GR, HNF4α and miR122-5p in the liver of HFD/STZ-induced T2DM mice. (**G**) Protein levels of HNF4α, G6Pase and PEPCK in the liver of HFD/STZ-induced T2DM mice were detected by western blot assay and quantified in (**H**). All results were presented as mean ± S.E.M (*P<0.05, **P<0.01 and ***P<0.001).

Since we have determined that FX5 treatment decreased gluconeogenesis in mouse primary hepatocytes and improved PTT in T2DM mice, we next investigated its capability in inhibiting gluconeogenic genes in the liver tissues of T2DM mice by RT-PCR. The results (Figure 6A for *db/db* mice, Figure 6E for HFD/STZ-induced T2DM mice) indicated that FX5 treatment restrained the mRNA levels of G6Pase and PEPCK in T2DM mice. Meanwhile, we also evaluated the influence of FX5 on the protein levels of G6Pase and PEPCK in the liver tissues of T2DM mice by western blot, and the results demonstrated that FX5 treatment (Figure 6C, 6D for *db/db* T2DM mice, Figure 6G, 6H for HFD/STZ induced T2DM) decreased the protein levels of these two gluconeogenesis genes.

Together, all results demonstrated that FX5 inhibited gluconeogenesis in T2DM model mice.

### FX5 treatment inhibited GR/HNF4α/miR122-5p signaling in T2DM mice

Given that FX5 reduced gluconeogenesis via GR/HNF4α/miR122-5p signaling pathway in primary hepatocytes, we verified such FX5-mediated regulation in the liver tissues of T2DM mice. Western blot (Figure 6C, 6D, Figure 6G, 6H) and quantitative RT-PCR (Figure 6B, 6F) results indicated that FX5 treatment suppressed protein level of HNF4α, mRNA levels of GR and HNF4α, as well as expression level of miR122-5p in *db/db* or HFD/STZ induced T2DM mice.

Thus, all results indicated that FX5 treatment repressed GR/HNF4α/miR122-5p pathway.

## DISCUSSION

Gluconeogenesis is regulated by a series of key enzymes including G6Pase and PEPCK [4], and targeting G6Pase or PEPCK has been determined an effective strategy for improving hyperglycemia in T2DM [2]. GR as a transcription regulator is tightly implicated in gluconeogenesis regulation. It binds to GRE in the promoters of G6Pase and PEPCK, and the activity of GR is regulated by glucocorticoids instead of glucagon or insulin [41, 43]. Currently, accumulating evidence has supported that targeting GR is a promising strategy for anti-T2DM drug discovery, although the underlying mechanism remains obscure. Here, we determined that small molecule FX5 as a novel GR inhibitor effectively inhibited gluconeogenesis and improved hyperglycemia in T2DM model mice. Moreover, the result that long-term treatment of FX5 caused low level of insulin in serum (Supplementary Figure 4E, 4F) indicated that FX5 treatment ameliorated the insulin sensitivity of T2DM mice. Our results have thus highlighted the potential of FX5 in the treatment of T2DM.

As indicated in the published results, GR may regulate gluconeogenic genes through PGC-1α and FoxO1 signaling [44, 45]. However, we found that FX5 treatment had no impacts on either of these two signaling in the liver tissues of T2DM model mice (Supplementary Figure 2A–2D). Additionally, GR could also activate its downstream gene GC-induced leucine zipper (GILZ) to impair inflammatory factor gene transcription [46, 47], we here found that FX5 treatment rendered no effects on mRNA level of GILZ in T2DM model mice (Supplementary Figure 2E, 2F). Thus, our findings have revealed a new mode for FX5 in the regulation of GR-mediated gluconeogenic genes and GR downstream genes. Since selective GR modulator (SGRM) discovery strategy is generally focused on GR downstream anti-inflammation gene rather than gluconeogenic gene [18] causing side effects for GR modulator, our results might hopefully provide a new approach for designing GR modulator with better drugability.

HNF4α as a transcriptional regulator directly binds to the promoters of G6Pase and PEPCK, and GR regulates HNF4α gene expression [41, 48]. Targeting the inhibition of HNF4α has become a novel strategy for T2DM treatment, and several HNF4α-targeted compounds such as flavonoid luteolin and 4-nitro-6-hydroxyflavone have been determined to be able to improve glucose homeostasis [49, 50]. Here, we found that FX5 repressed HNF4α gene in the liver tissues of T2DM model mice. Interestingly, FX5 could not promote HNF4α degradation (Supplementary Figure 1H, 1I), different from the case for 4-nitro-6-hydroxyflavone in the suppression of HNF4α [50]. Additionally, circulating miR122 is closely related to the risk of metabolic syndrome and type 2 diabetes [51], and miR122-5p expression was upregulated in the liver of HFD/STZ-induced diabetic mice [26, 27]. HNF4α was reported to positively regulate miR122 [27]. Here, we also determined that FX5 suppressed gluconeogenic genes involving miR122-5p regulation. Although the detailed underlying mechanism remained obscure regarding the regulation of HNF4α against miR122-mediated gluconeogenic genes, there has been published report that miRNAs may bind to the 3’-untranslated regions of target mRNAs to modify gene expression at the post-transcriptional level thereby resulting in mRNA transcript degradation or repression [52, 53]. Thus, we tentatively hypothesized that FX5-mediated miR122-5p may also repress the mRNA expressions of G6Pase and PEPCK through directly suppressing or degrading their transcriptions, while further validation remained to be addressed.

Finally, it was noticed that hypothalamic pituitary adrenal (HPA) axis is hyperactive in T2DM patients and model mice [54, 55]. HPA axis is a neuroendocrine system controlling GC section via a cascade of hormonal events [56]. Long-term treatment of GR antagonist was reported to cause undesirable side effects including adrenal insufficiency and activation of HPA axis [20]. Currently, several non-steroidal GR antagonists have been reported to exhibit reduced activity against HPA axis while maintain anti-diabetic efficiency [57]. In this case, much work is needed to intensively examine the regulation of FX5 against HPA axis in T2DM mice during the long-term administration.

**Figure 7:**
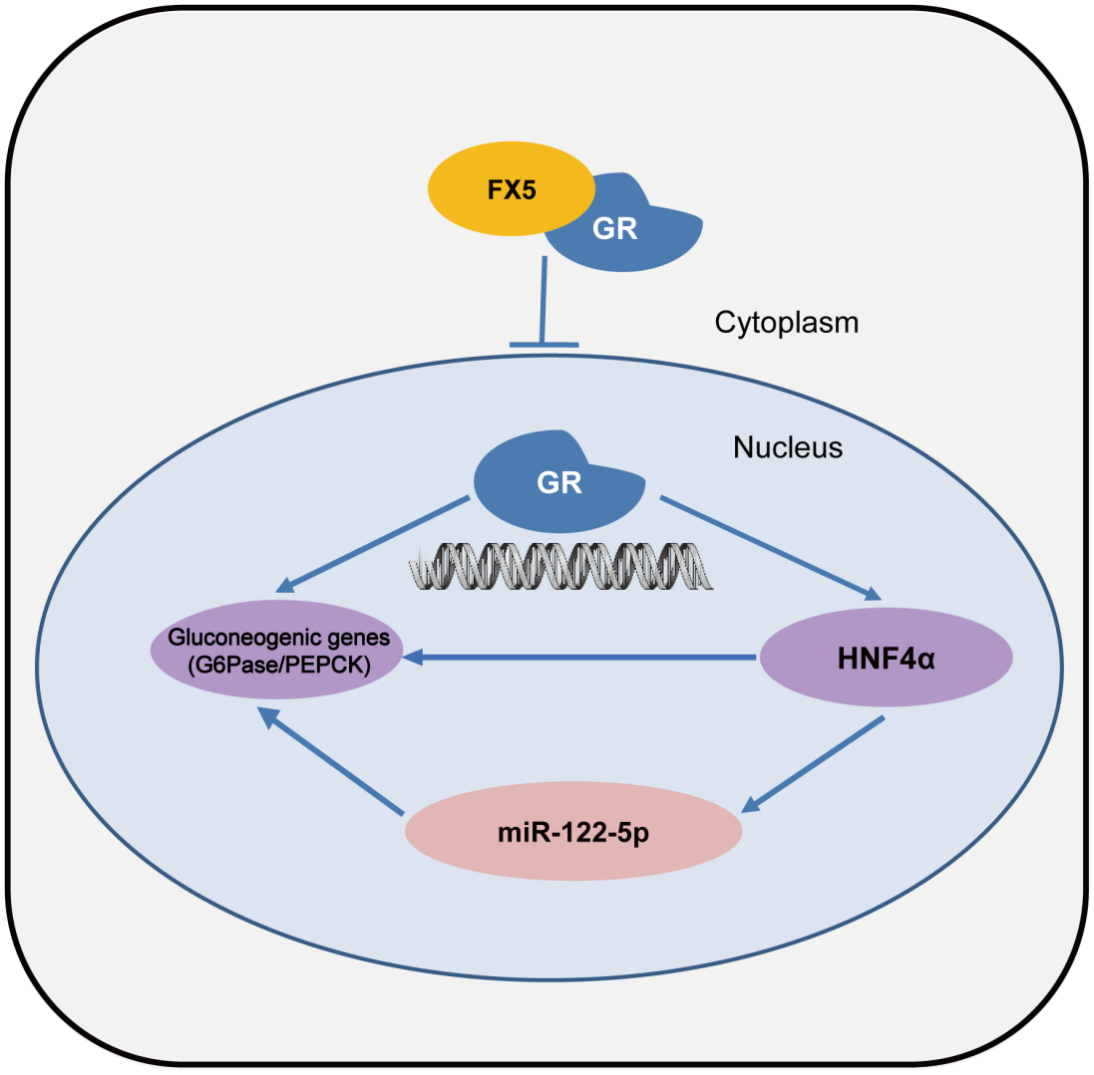
**A proposed model describing the regulation of FX5 against gluconeogenesis and glucose homeostasis.** FX5 suppressed gluconeogenetic genes G6Pase and PEPCK, and improved hyperglycemia in T2DM mice by targeting GR and involving GR/HHNF4α and GR/HNF4α/miR122-5p pathways.

In summary, we determined that small molecule FX5 efficiently improved hyperglycemia in T2DM mice. The underlying mechanism has been intensively investigated. As illustrated in Figure 7, FX5 alleviated gluconeogenesis either directly by antagonizing GR or indirectly through GR/HNF4α/miR-122-5p signaling pathway. Our work has presented a new mode for GR antagonist in the regulation of gluconeogenesis, which may help design new generation of GR modulators against T2DM and highlight the potential of FX5 in the treatment of this disease.

## MATERIALS AND METHODS

### Materials

Dimethyl sulfoxide (DMSO), Tween-80, streptozocin (STZ), 3-(4,5-dimethyl-2-thiazolyl)-2,5-diphenyl-2-H-tetrazolium bromide (MTT), sodium lactate, sodium pyruvate, dexamethasone (Dex) and mifepristone (MIFE) were purchased from Sigma-Aldrich (St. Louis, Missouri, USA). Glucagon and BI6015 were from Toronto Research Chemicals (Toronto, Canada). Cycloheximide (CHX) was obtained from MedChemExpress (Monmouth Junction, New Jersey, USA). All reagents for cell culture were from Gibco (Grand Island, New York, USA) and for RNA isolation and PCR were purchased from Takara (Dalian, Liaoning, China). Antibodies of G6Pase, PEPCK, GR, Histone H3 and GAPDH were purchased from Cell Signaling Technology (CST, Beverly, Massachusetts, USA). 11β-hydroxysteroid dehydrogenase 1 (11β-HSD1) and HNF4α antibodies were from Abcam (Cambridge, UK). 11β-hydroxysteroid dehydrogenase 1 (11β-HSD1) and HNF4α antibodies were from Abcam (Cambridge, UK).

Plasmids of pCI-nGFP-C656G, pRL-SV40 and pUAS-TK-Luc were kindly donated by Dr. Gordon Hager (National Cancer Institute, National Institutes of Health), Dr. J Larry Jameson (Department of Medicine, Northwestern Memorial Hospital) and Dr. Daniel P. Kelly (School of Medicine, Washington University). Other plasmids were available in our own lab.

### Cell cultures

Human embryonic kidney HEK293T cells were purchased from ATCC (Manassas, Virginia, USA) and cultured in Dulbecco’s minimum essential medium (DMEM supplemented with 10% FBS, 100 U/mL penicillin and 100 μg/mL streptomycin) at 37° C in 5% humidified CO_2_ incubator.

U2OS/GR-GFP cells were obtained from Bioimage (Denmark) and cultured in DMEM (supplemented with 10% FBS, 100 U/mL penicillin and 100 μg/mL streptomycin) at 37° C in 5% humidified CO_2_ incubator.

Primary mouse hepatocytes were isolated from male C57/BL6 mice according to the previously reported method and cultured at 37° C in 5% humidified CO_2_ incubator [58]. After cells were adhered to the plate, the medium was changed into MEM (supplemented with 10% FBS, 100 U/mL penicillin and 100 μg/mL streptomycin).

### Mammalian one-hybrid and transactivation assays

Mammalian one-hybrid assay was carried out to investigate the regulation of FX5 against GR-LBD (LBD, ligand binding domain) according to the reported approach [33]. Briefly, HEK293T cells were seeded at a density of 5×10^4^ cells/well in 48-well plates and cultured overnight, and then co-transfected with plasmids of pCMX-Gal4-GR-LBD, pUAS-TK-luc and pRL-SV40 by calcium phosphate cell transfection kit (Beyotime, Haimen, Jiangsu, China). After 6 h, the cells were incubated with Dex (10 nM, known GR agonist) and different concentrations of FX5 for 12 h. Finally, firefly and renilla luciferase activities were measured by dual-luciferase reporter assay system kit (Promega, Madison, Wisconsin, USA).

Mammalian transactivation experiment was conducted to detect the antagonistic effect of FX5 on GR transcriptional activity. In the assay, HEK293T cells were co-transfected with the plasmids of pCI-nGFP-GR (C656G), pGL3-GRE-Luc and pRL-SV40. After 6 h, the cells were incubated with Dex (10 nM) and different concentrations of FX5 for 12 h. Finally, firefly and renilla luciferase activities were detected by dual-luciferase reporter assay system kit.

### Nuclear translocation assay

The assay was performed according to the published approach [33]. U2OS/GR-GFP cells were seeded at a density of 1×10^4^ cells/well in 96-well plates. Different concentrations of FX5 were added into the medium and incubated for 5.5 h, and cells were then incubated with 5 nM Dex for another 0.5 h. Finally, the cells were immobilized with 4% paraformaldehyde, and nucleus was dyed with Hoechst 33342 (Solarbio, Beijing, China). Fluorescence image was obtained using Leica DMi8 fluorescence microscope (Leica Microsystems, Nussloch, Germany). Ratio of fluorescence intensity in nucleus to cytoplasm was calculated by ImageJ software.

### Nuclear and cytoplasmic protein extraction

The experiment was conducted based on the previously published report [58]. Briefly, mouse primary hepatocytes were seeded into 10-cm cell culture dish. Dex (10 nM) and different concentrations of FX5 were added into the medium and incubated for 6 h. Then, the cells were washed twice with PBS and harvested, nuclear and cytoplasmic proteins were extracted by the nucleus and cytoplasm extraction kit (Beyotime). Protein concentration was detected by BCA protein assay kit (Beyotime).

### Western blot assay

The assay was conducted based on the published method [59]. Briefly, mouse primary hepatocytes or the liver tissues of mice were harvested, and targeted protein in the lysate was then separated by SDS-PAGE and transferred into nitrocellulose membrane (GE Healthcare, Waukesha, Wisconsin, USA). Primary antibodies were incubated with membrane at 4° C overnight, and secondary antibodies were then incubated at room temperature for 2 h. Chemiluminescence signals were collected by Tanon-5200 Chemiluminescent Imaging System (Tanon Science and Technology Co, Shanghai, China).

### Glucose output assay

The assay was conducted according to the previously published approach [60]. Briefly, mouse primary hepatocytes were cultured with MEM in 48-well plate, followed by incubation with glucagon (10 nM) or Dex (10 nM) overnight. Then, the cells were incubated with FX5 (1, 5 and 10 μM) for additional 6 h in glycogenetic medium (glucose-free and phenol red-free DMEM, pH 7.4, containing 20 mM sodium lactate, 2 mM sodium pyruvate and 10 nM glucagon). Finally, concentration of glucose in the medium was testified by glucose assay kit (Nanjing Jiancheng Bioengineering Institute, Nanjing, Jiangsu, China) and total protein concentration was measured to normalization by BCA protein assay kit (Beyotime).

### MTT assay

The experiment was performed according to the published method [61]. Briefly, mouse primary hepatocytes were seeded in 48-well plate and cultured in MEM overnight. Cells were then incubated with different concentrations of FX5 for 24 h, and 0.5 mg/mL MTT was added into the medium and incubated for additional 4 h. Finally, the formed formazan crystals were dissolved in DMSO for 10 min and OD (490 nm) was recorded by SpectraMax i3x reader (Molecular Devices, Sunnyvale, California, USA).

### Transfection of RNA interference and overexpression plasmids

Mouse primary hepatocytes were cultured in 12-well plates with serum free MEM medium. 50 nmol *si*RNA-GR (GenePharma, Shanghai, China), HNF4α overexpression plasmid (GenePharma, Shanghai, China), miR122-5p mimic or (Ribobio, Guangzhou, China) *si*-miR122-5p (Qiangen, Cambridge, USA) was dissolved in 100 μL Opti-MEM, and mixed with transfection reagent (4.5 μL Lipofectamine 2,000 was dissolved in 100 μL Opti-MEM) for 15 min. After 6 h incubation, it was replaced by complete MEM. After 48 h culture, the compounds were added and incubated in the cells for 6 h with or without Dex.

### RNA isolation and quantitative real-time PCR

Total RNA in mouse primary hepatocytes and the liver tissues of mice were extracted by RNAiso according to the previous report [62]. Then, 1 μg total RNA was reverse-transcribed into cDNA using PrimeScript RT reagent kit. Finally, mRNA levels of different genes were quantified by quantitative real-time PCR using SYRB Premix Ex Taq kit and normalized to GAPDH. Micro RNA was performed using Mir-X miRNA First-Strand Synthesis kit. Micro RNA levels were quantified by qRT-PCR using Mir-X miRNA qRT-PCR TB Green Kit normalized to U6 according to the manufacturer’s instructions. All reagents were purchased from Takara (Dalian, Liaoning, China). The primers were synthesized by Shenggong Biotechnology (Shanghai, China) as follows (Supplementary Table 1).

### Animal experiment

All animals were received humane care and animal-related protocols were approved by the Institutional Animal Care and Use Committees at Nanjing University of Chinese Medicine.

6-week-old *db/db* male mice (BKS-Leprem2Cd479/Nju) and male C57BL/6J mice were obtained from Nanjing Biomedical Research Institute of Nanjing University and cultured in SPF room. *db/db mice* were used for experiments at 8 weeks of age after 2-week acclimation. HFD/STZ-induced T2DM mice were built according to the published method [58, 63]. In brief, 6-week-old C57BL/6 male mice were fed with standard diet for 1 week, and then fed with high-fat diet (58% fat, 17% carbohydrate and 25% protein) for 4 weeks. After 8 h fasting, 100 mg/kg STZ was administered by intraperitoneal injection to animals feeding with high fat diet for establishing a diabetic model.

*db/db* mice (n=10/group) or HFD/STZ-induced T2DM mice (n≥7/group) were divided into three groups based on the fasting blood glucose, and orally administrated with vehicle (2% DMSO, 5% Tween-80 and 93 % saline) or FX5 (30, 60 mg/kg/day) respectively for 5 weeks. Specifically, FX5 was at first diluted with 2% DMSO, followed by the addition of 5% Tween-80 slowly until the compound was completely dissolved. Finally, 93% saline was added to the clear solution carefully after shaking, and the final solution was clear.

Plasma glucose concentration of mice after 6 h fasting was testified weekly, and glycated hemoglobin (HbA1c) level was measured by DCA Vantage analysis system (Siemens, Erlangen, Germany) after 5 weeks. Then, the livers were isolated and stored at -80° C after the mice were sacrificed.

### OGTT and PTT assays

Oral glucose tolerance test (OGTT) and pyruvate tolerance test (PTT) were assayed according to the published approaches [58]. Mice were fasted overnight, and 1 g/kg glucose was then orally administered (for OGTT assay) or 1.5 g/kg sodium pyruvate was given by intraperitoneal injection (for PTT assay). During these two assays, glucose concentration from the tail blood was measured at 0, 15, 30, 45, 60, 90, and 120 min after administration by ACCU-CHEK Active blood sugar system (Roche, Basel, Switzerland).

### Statistical analysis

Data were shown as mean ± S.E.M. The values were compared by analysis of variance (ANOVA) test among more than two groups by GraphPad Prism 7.0 Software (La Jolla, California, USA). Statistical significance was indicated by * (*P*<0.05), ** (*P*<0.01) and *** (*P*<0.001).

## Supplementary Material

Supplementary Figures

Supplementary Table 1
