# Supplementary material for: FX5 as a non-steroidal GR antagonist improved glucose homeostasis in type 2 diabetic mice via GR/HNF4α/miR-122-5p pathway
Source: Aging (Albany NY). 2020 Dec 9;13(2):2436–58. doi: 10.18632/aging.202275 (PMC7880398; doi:10.18632/aging.202275)
Supplement: Supplementary Figures [file aging-13-202275-s001.pdf]

SUPPLEMENTARY FIGURES

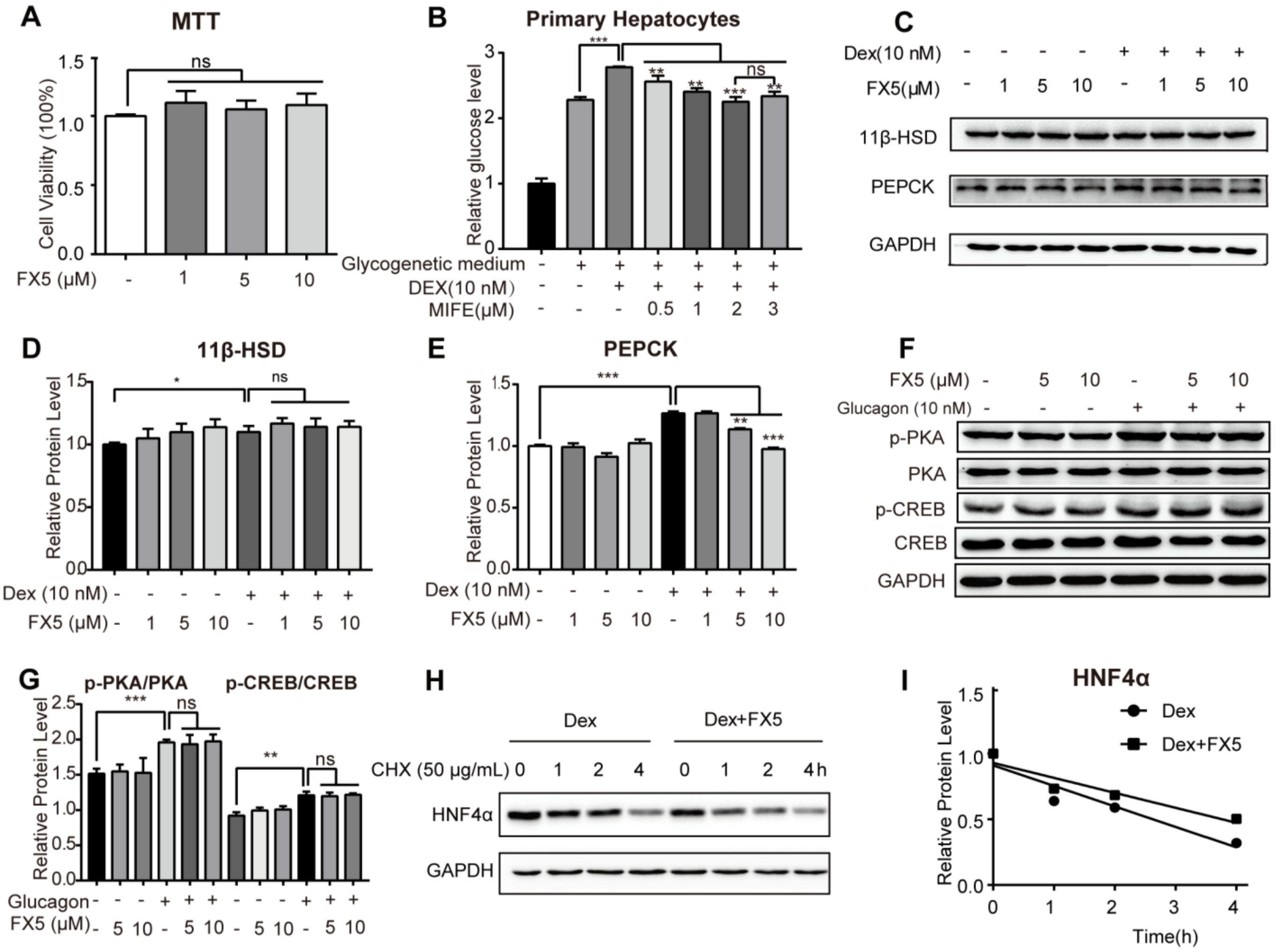

Supplementary Figure 1. FX5 regulated gluconeogenesis independent of repression of 11β-HSD protein level, regulation of PKA/CREB signaling or degradation of HNF4α in primary hepatocytes. (**A**) Mouse primary hepatocytes were treated with different concentrations of FX5 (1, 5, 10 μM) for 24 h, and cell viability was detected by MTT assay. (**B**) Primary hepatocytes were pretreated with glucagon (10 nM) and different concentration of mifepristone (MIFE) (0.5, 1, 2, 3 μM) for 12 h, and then cultured for another 6 h in glycogenetic medium with glucagon and MIFE. Finally, glucose level in the medium was measured. (**C**) Mouse primary hepatocytes were incubated with 10 nM Dex and different concentrations of FX5 (1, 5, 10 μM) for 6 h. Finally, protein levels of 11β-HSD, PEPCK and GAPDH were tested by western blot assay. Quantification of (**D**) 11β-HSD and (**E**) PEPCK protein levels, all results were normalized to GAPDH. (**F**) FX5 (5, 10 μM) with or without Dex (10 nM) was incubated in mouse primary hepatocytes for 6 h, and protein levels of PKA-CREB signaling pathway were detected by Western blot. (**G**) Quantification of p-PKA and p-CREB protein levels. (**H**)Mouse primary hepatocytes were pretreated with Dex (10 nM) and FX5 (10 μM) for 4 h, followed by incubation with 50 μg/mL CHX in medium for 0, 1, 2 and 4 h. Protein level of HNF4α was tested by western blot. (**I**) Quantification of HNF4α protein level normalized to GAPDH. Horizontal axis stands for the incubated time with CHX, and vertical axis for HNF4α/GAPDH.

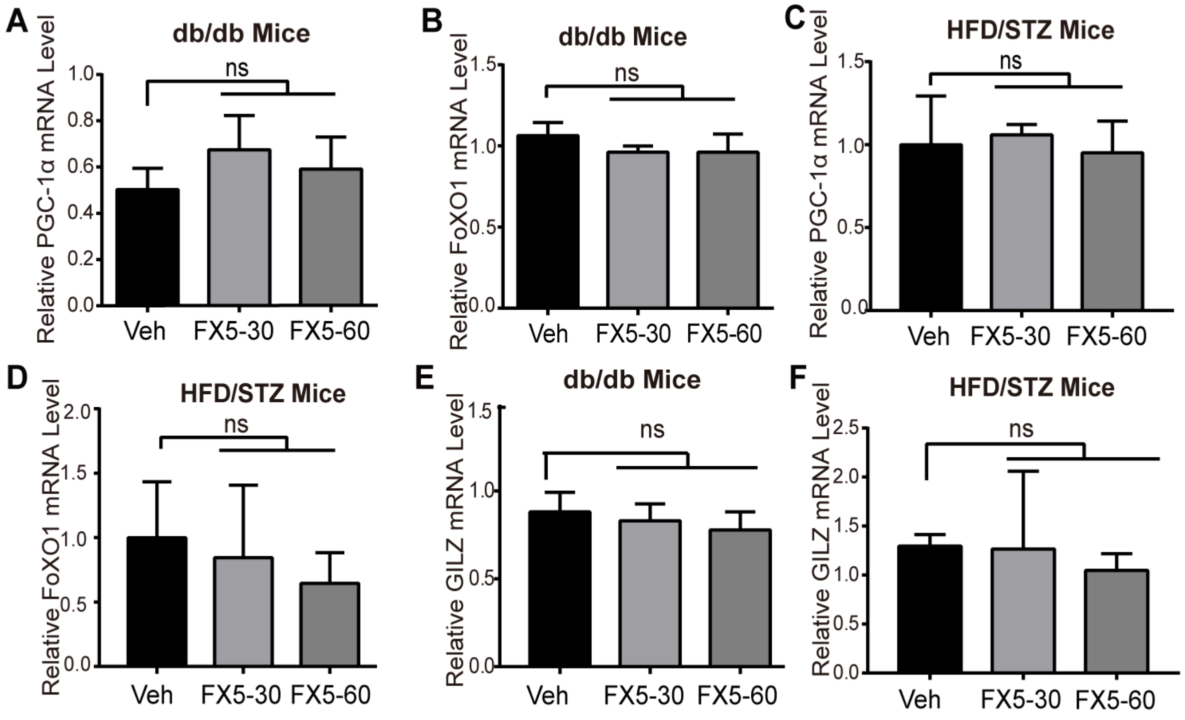

Supplementary Figure 2. FX5 had no effects on the mRNA levels of PGC-1α, FOXO1 or GILZ in the liver tissues of T2DM mice. mRNA levels of PGC1α and FoxO1 in liver tissue of (**A**, **B**) *db/db* mice and (**C**, **D**) HFD/STZ-induced T2DM mice were measured by quantitative RT-PCR assay, and results were normalized to GAPDH. mRNA level of GILZ was detected in liver tissue of (**E**) *db/db* and (**F**) HFD/STZ-induced T2DM mice. All results were presented as mean ± S.E.M (**P*<0.05, ***P*<0.01 and ****P*<0.001).

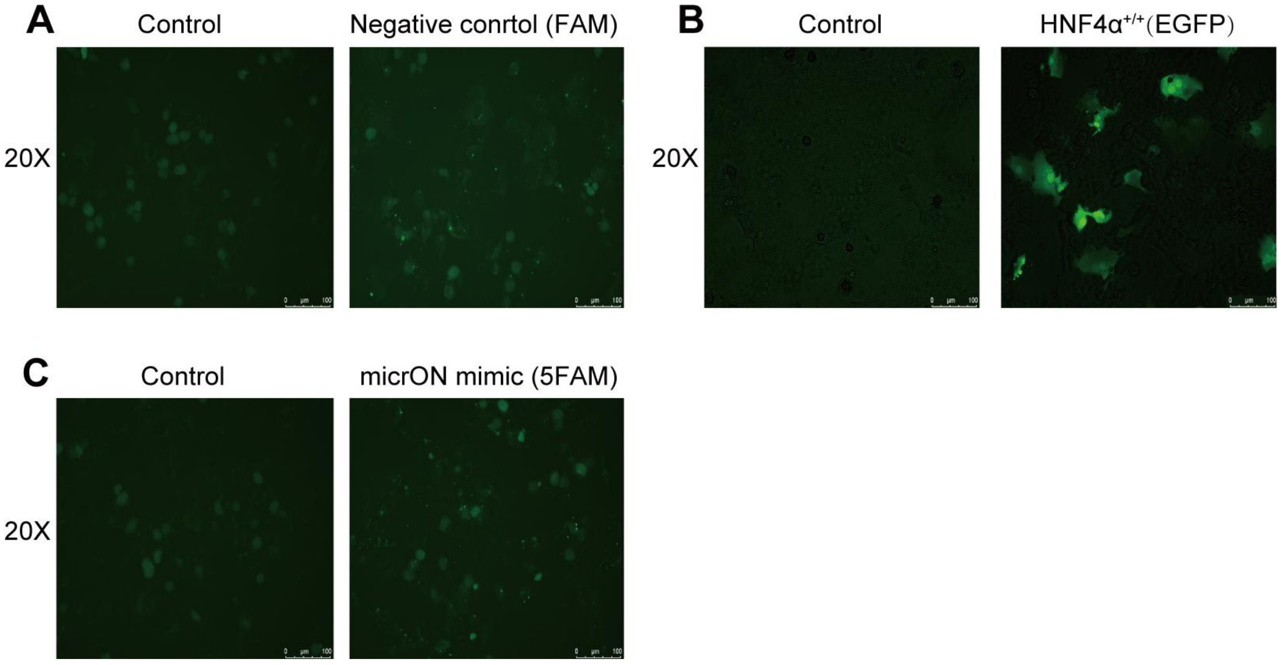

Supplementary Figure 3. Transfection efficiency of plasmids in primary hepatocytes. Primary hepatocytes were transduced with (**A**) small interference negative control (FAM), (**B**) HNF4α overexpression plasmid (EGFP) and (**C**) micrON mimic NC #22 (FAM) for 48h, and images were taken using a fluorescence microscope.

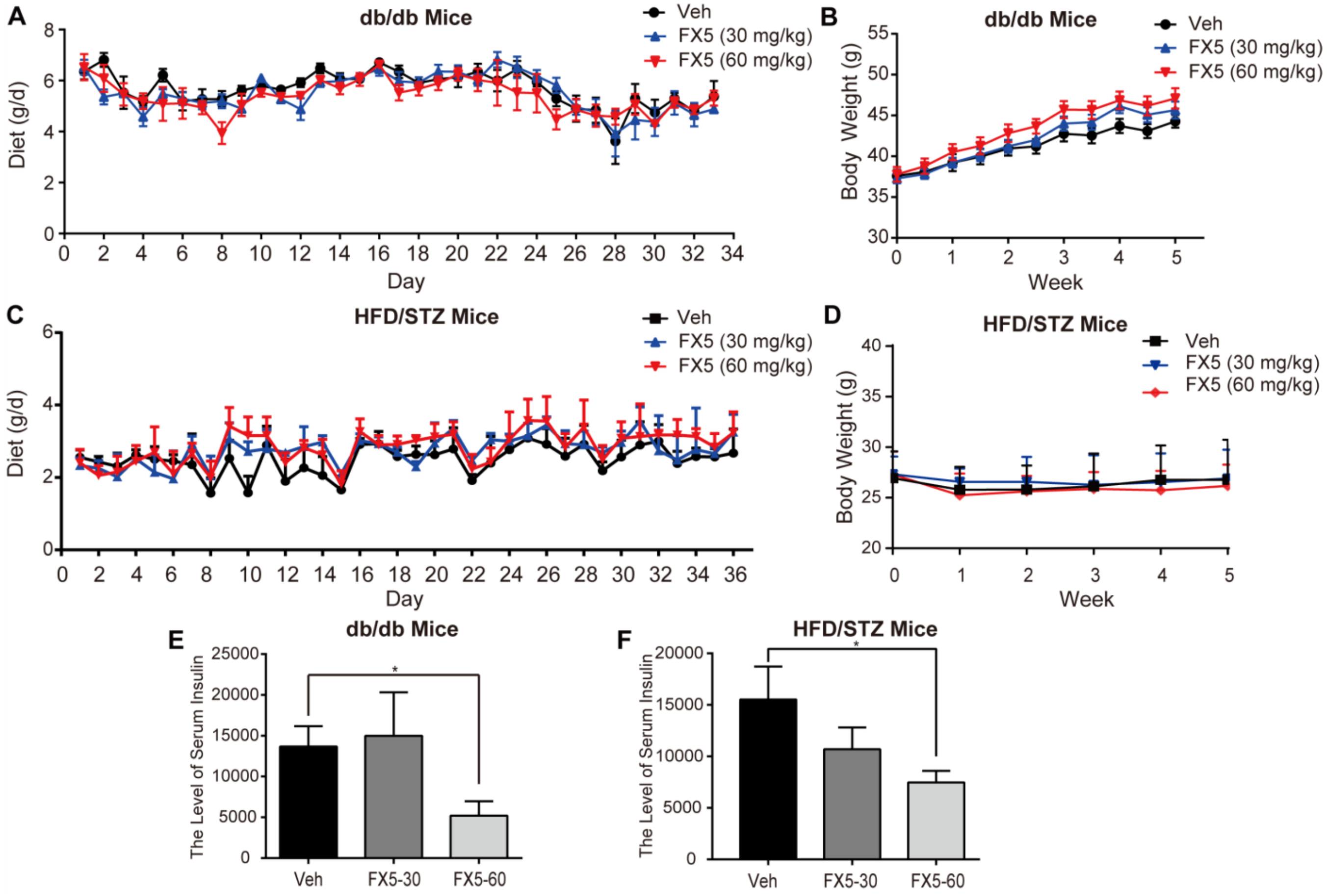

Supplementary Figure 4. FX5 treatment had no effects on food intake or body weight but decreased serum insulin level of *db/db* and HFD/STZ induced diabetic mice. (**A**) Food intake and (**B**) body weight of *db/db* mice during the treatment. (**C**) Food intake and (**D**) body weight of HFD/STZ mice during the treatment. (**E**) Serum insulin level of *db/db* mice. (**F**) Serum insulin level of HFD/STZ mice.
