# Supplementary material for: FX5 as a non-steroidal GR antagonist improved glucose homeostasis in type 2 diabetic mice via GR/HNF4α/miR-122-5p pathway
Source: Aging (Albany NY). 2020 Dec 9;13(2):2436–58. doi: 10.18632/aging.202275 (PMC7880398; doi:10.18632/aging.202275)
Supplement: Supplementary Table 1 [file aging-13-202275-s002.pdf]

SUPPLEMENTARY TABLE

Supplementary Table 1. List of mouse primer sequences for RT-PCR assay.

Primer	Forward	Reverse
G6Pase	CGACTCGCTATCTCCAAGTGA	GTTGAACCAGTCTCCGACCA
PEPCK	CTGCATAACGGTCTGGACTTC	CAGCAACTGCCCGTACTCC
HNF4α	GGCAATGACACGTCCCCATCTG	GCCTGCAGTAACGACACTGGTTCC
FoxO1	CCCAGGCCGGAGTTTAACC	GTTGCTCATAAAGTCGGTGCT
PGC-1α	TTCTGGGTGGATTGAAGTGGTG	TGTCAGTGCATCAAATGAGGGC
GILZ	CAGCTGCACAATTTCTC	CATCAGGTGGTTCTTCAC
GR	TGCTATGCTTTGCTCCTGATCTG	TGTCAGTTGATAAAACCGCTGC
GAPDH	ACAGCAACAGGGTGGTGGAC	TTTGAGGGTGCAGCGAACTT
miR122-5p	TGGAGTGTGACAATGGTGTTTG	
